# Potential Effect of L-Carnitine on the Prevention of Myocardial Injury after Coronary Artery Bypass Graft Surgery

**Published:** 2015-04-03

**Authors:** Farzaneh Dastan, Azita Hajhossein Talasaz, Mojtaba Mojtahedzadeh, Abbasali Karimi, Abbas Salehiomran, Payvand Bina, Arash Jalali, Zahra Aghaie

**Affiliations:** 1 *School of Pharmacy, Tehran University of Medical Sciences, Tehran, Iran. *; 2 *Tehran Heart Center, Tehran University of Medical Sciences, Tehran, Iran.*

**Keywords:** Coronary artery bypass graft, CK-MB, Troponin T, L-carnitine

## Abstract

**Background:** L-carnitine has been demonstrated to confer cardiac protection against ischemia reperfusion injury in animals. This study evaluates the effects of L-carnitine administration on cardiac biomarkers after coronary artery bypass graft (CABG) surgery.

**Methods:** One hundred thirty-four patients undergoing elective CABG surgery, without a history of myocardial ischemia or previous L-carnitine treatment, were enrolled and randomly assigned to an L-carnitine group ([n = 67], 3000 mg/d, started 2 days preoperatively and continued for 2 days after surgery) or a control group (n = 67). CK-MB (creatine kinase, muscle-brain subunits) and troponin T (TnT) levels were assessed in all the patients before surgery as baseline levels and at 8 and 24 hours postoperatively.

**Results: **Our study included 134 patients (99 [73.8%] males) at a mean ± SD age of 59.94 ± 8.61 years who were candidates for CABG and randomized them into control or L-carnitine groups. The baseline demographic characteristics, including age (60.01 ± 9.23 in the L-carnitine group vs. 59.88 ± 7.98 in the control group) and sex (54 [80.6%] in the L-carnitine group vs. 45 [67.2%] in the control group) did not show any significant differences (p value=0.93 and 0.08, respectively). Patients in the L-carnitine group had lower levels of CK-MB (mean ± SD, 25.06 ± 20.29 in the L-carnitine group vs. 24.26 ± 14.61 in the control group), but the difference was not significant (p value = 0.28). TnT levels also showed no significant differences between the two groups (399.50 ± 378.91 in the L-carnitine group vs. 391.48 ± 222.02 in the control group; p value = 0.34).

**Conclusion:** In this population of intermediate- to high-risk patients undergoing CABG surgery, L-carnitine did not reduce CK-MB and TnT levels.

## Introduction

One of the most useful treatments for relieving angina and improving survival and quality of life in patients with multivessel coronary artery disease is coronary artery bypass graft (CABG) surgery.^1^ Although CABG mortality has reduced over time, serious complications such as myocardial infarction (MI), recurrent angina, ventricular failure, serious arrhythmias, renal insufficiency, and stroke remain to limit this surgical method, particularly in high-risk patients.^2^^-^^7^ Ischemia-reperfusion injury is known to be one of the major mechanisms involved in such complications.^8^ If outcomes among CABG patients are to be improved, development of better strategies to reduce ischemia-reperfusion injury is imperative.

Carnitine is a natural amino acid which is necessary for the transport of long-chain fatty acids from the cell cytoplasm to the mitochondrial matrix, where β-oxidation of fatty acids and thus, adenosine triphosphate (ATP) production occur.^9^^, ^^10^ Carnitine is also reported to be an essential cofactor which can reduce ischemia-reperfusion injury in the myocardium. Recent studies have indicated its effectiveness in the recovery of post-ischemic cardiac function, confirmed by an experimental model of myocardial ischemia-reperfusion. These studies showed that carnitine could significantly reverse mechanical dysfunction during both myocardial ischemia and reperfusion.^9^^-^^11^ Carnitine deficiency is the major cause of impaired metabolism, which mainly results in cardiomyopathy.^12^ Therefore, the administration of carnitine as a treatment for the deficiency is supported by a substantial amount of evidence.^13^^-^^15^ Moreover, carnitine in the myocardium avoids fatty acid gathering and lactic acid production, resulting in the enhancement of myocardial function.^16^

Creatine kinase-MB (CK-MB) and cardiac troponin T (cTnT) are important diagnostic tools in the evaluation of major and minor myocardial injuries.^17^^, ^^18^ Several methods and therapeutic agents have been assessed for their ability to decline cardiac complications in the perioperative setting.^19^ L-carnitine has been demonstrated to provide cardiac protection against ischemia-reperfusion injury in a number of animal models.^20^ However, very few studies have examined the clinical effect of L-carnitine on cardiac biomarkers.

The objective of this study was to determine the effects of L-carnitine administration on cardiac biomarkers (CK-MB and cTnT) after coronary artery bypass surgery.

## Methods

The study was registered as RCT201404268698N13 at the Iranian Registry of Clinical Trials and was approved by the Ethics Committee of Tehran Heart Center (THC), a referral hospital. Patients eligible for CABG surgery were enrolled between April and December 2013 at THC. Those with the following conditions were excluded: history of any preoperative supraventricular arrhythmia; concomitant valve surgery; history of antiarrhythmic drugs except for beta blockers and calcium channel blockers; history of seizure or epilepsy; history of hypersensitivity to L-carnitine; chronic liver insufficiency (liver enzyme levels more than 3 times the upper limit of normal); chronic kidney disease (stages IV & V of CKD); ejection fraction of less than 30%; recent MI (in the previous 4 weeks); uncontrolled hypertension; congestive cardiac failure; atrioventricular or left bundle branch block; history of anti-inflammatory medications for at least 2 weeks before admission; and hypothyroidism. 

Ultimately, 134 patients were included in the study population and randomly divided into two groups: the L-carnitine group, who were administered an oral solution of L-carnitine (So.Se.PHARM, Italy) 1 g, 3 times a day from 48 hours before the planned surgery to 48 hours afterward, and the control group, who did not receive L-carnitine. All the patients signed a consent form before surgery. 

Randomization was performed using the permuted random block. The randomization is described in the consort flowchart (Figure 1). Chest X-ray, echocardiography, and adverse reactions associated with L-carnitine, as well as the complications were followed up in both groups. CK-MB and cTnT levels were assessed in all the selected patients before surgery and at 8 and 24 hours postoperatively. The patients' demographics, past medical history, prior medication use, cardiac catheterization results, presenting cardiac syndrome, surgical data (i.e. type of surgery, number of bypass grafts, bypass/ ischemic times, and intraoperative complications), postoperative data (i.e. Intensive Care Unit length of stay and results of postoperative electrocardiography), postoperative outcomes, and presence of complications were collected. All the patients were followed up through to the end of their hospitalization for the assessment of the outcomes. 

Blood samples were cooled to 4 ^°^C and centrifuged at 3000 rpm for 10 minutes at 4 ^°^C. Serum was stored at −70 ^°^C until assay. The levels of MB isoenzyme of creatine kinase (CK-MB) were calculated using Elecsys CK-MB STAT Immunoassays (Roche Diagnostics Corporation, Indianapolis, Indiana) on an Elecsys 1010 platform (Roche Diagnostics Corporation). Also, cTnT was measured quantitatively using a one-step enzyme immunoassay based on electrochemiluminescence technology (Elecsys 2013; Roche, Mannheim, Germany). The lower detection limit of this assay is 0.01 µg/L (normal < 0.1 µg/L). 

Statistical analysis was performed using the SPSS 18.0 statistical software package. All p values < 0.05 were considered statistically significant. The Kolmogorov-Smirnov test was applied to determine the normality of data distribution. The nonparametric test (Man-Whitney) was then conducted to evaluate the statistically significant variables for the non-normal distributed data. The data with normal distribution were subjected to an independent t-test.

## Results

The patients’ demographic data are described in Table 1. There were no significant differences between the two study groups in age, gender, body mass index, history of MI, concomitant diseases (i.e. diabetes, hypertension, hyperlipidemia, and cerebrovascular accident), preoperative medications, and the number of the involved vessels as most of the patients had three-vessel disease.

Serum CK-MB levels in both groups were assessed. The baseline CK-MB levels (before surgery) showed no significant differences between the two groups (1.56 ± 0.77 ng/mL in the L-carnitine group vs. 1.47 ± 0.72 ng/mL in the control group; p value = 0.47).

Comparison of the CK-MB level changes at baseline and at 8 hours post CABG between the L-carnitine and control groups showed no significant differences (p value = 0.08). The CK-MB level changes at baseline and at 24 hours post CABG (p value = 0.4) and the ones at 8 hours and 24 hours postoperatively (p value = 0.19) also demonstrated the same results as there were no significant differences in these levels between the L-carnitine and the control groups (Table 2).

The baseline cTnT levels (before surgery) demonstrated no significant differences between the two groups (11.80 ± 7.10 µg/L in the L-carnitine group vs. 12.11 ± 8.09 µg/L in the control group; p value = 0.93). The analysis of the cTnT level changes at baseline and at 8 hours post CABG also showed no significant differences between the two study groups (p value = 0.07). The cTnT level changes at baseline and at 24 hours post surgery and the ones at 24 hours and 8 hours postoperatively were also found to have no significant differences between the L-carnitine group and the control one (p value = 0.34 and p value = 0.11, respectively) (Table 3).

The post-CABG characteristics of the groups are depicted in Table 4. Renal failure, cardiac arrest, aortic dissection, and liver function impairment were not observed. Redo for bleeding was not performed. Stroke and coma were reported in one patient, and prolonged ventilation (more than 21 days) was observed in 2 patients (both in the control group). Two cases of cardiac tamponade and one case of cerebrovascular accident were detected in the L-carnitine group.

**Table 1:** Comparison of the patients’ baseline characteristics and drug history*

	L-Carnitine Group (N=67)	Control Group (N=67)	P value
Age (y)	60.01±9.23	59.88±7.98	0.93
Male	54 (80.6)	45 (67.2)	0.08
BMI (kg/m^2^)	28.16±4.55	27.10±4.13	0.16
Diabetes Mellitus	23 (34.3)	28 (41.8)	0.37
Hypertension	42 (62.7)	40 (59.7)	0.72
MI	12 (18.2)	15 (22.4)	0.55
Hyperlipidemia	35 (52.2)	32 (47.8)	0.60
Smoking history	6 (9.0)	4 (6.0)	0.74
CVA	6 (9.0)	2 (3.0)	0.15
LVEF (%)	45.37±8.80	42.84±10.20	0.18
3VD	51 (76.1)	54 (81.8)	0.35
β- blocker	66 (98.5)	66 (98.5)	1.00
Ca^2+^ Channel Blocker	10 (14.9)	7 (10.6)	0.46
ACE/ARB	53 (79.1)	54 (80.6)	0.83
Aldosterone Antagonist	3 (4.5)	8 (11.9)	0.12
Nitrate	57 (85.1)	55 (82.1)	0.64
Statin	67 (100)	67 (100)	1.001

BMI, Body mass index; MI, Myocardial infarction; CVA, Cerebrovascular accident; LVEF, Left ventricular ejection fraction; 3VD, Three-vessel disease; ACE/ARB, Angiotensin-converting enzyme inhibitor/ Angiotensin receptor blocker

* Data are presented as mean±SD or n (%)

**Table 2:** Comparison of the patients’ cardiac biomarkers, creatine kinase and troponin T levels*

	L-Carnitine Group	Control Group	P value
CK-MB1 (ng/ml)	1.56±0.77	1.47±0.72	0.47
CK-MB2 (ng/ml)	24.25±14.60	25.05±20.29	0.28
cTnT1 (µg/L)	11.80±7.10	12.11±8.09	0.93
cTnT2 (µg/L)	391.48±222.02	399.49±378.91	0.34

CK-MB1, Baseline creatine kinase; CK-MB2, Peak level of creatine kinase between 8 and 24 hours post surgery; cTnT1, Baseline cardiac troponin T; cTnT2, Peak level of cardiac troponin T between 8 and 24 hours post surgery

* Data are presented as mean ± SD

**Table 3:** CK-MB and cTnT level changes between the L-carnitine and control groups

	Median	Interquartile Range	P value
CK-MB 8h-Baseline (ng/ml)	15.53	10.18±23.89	0.08
CK-MB 24h-Baseline (ng/ml)	8.03	3.62±13.16	0.41
CK-MB 24h-8h (ng/ml)	-7.58	-12.23± -1.50	0.19
cTnT 8h-Baseline (µg/L)	284.10	161.67±435.24	0.07
cTnT 24h-Baseline (µg/L)	158.68	80.27±298.33	0.34
cTnT 24h-8h (µg/L)	-97.10	-179.00± -22.60	0.11

CK-MB, Creatine kinase MB enzyme; cTnT, Cardiac troponin T; 8h-Baseline, Difference between the baseline level of the biomarker and that 8 hours post surgery; 24h-Baseline, Difference between the baseline level of the biomarker and that 24 hours post surgery; 24h-8h, Difference between the biomarker levels at 24 hours and 8 hours post surgery

**Table 4:** Comparison of the patients’ postoperative characteristics^*^

	L-Carnitine Group (N=67)	Control Group (N=67)	P Value
CPB time (min)	42.11±21.16	38.18±20.22	0.43
ACC time (min)	20.60±15.59	22.11±17.42	0.11
Prolonged mechanical ventilation	0	2 (3.0)	0.50
Inotrope usage	8 (12.5)	8 (12.5)	0.95
Blood product usage	10 (15.6)	17 (27.4)	0.11
Cardiac tamponade	2 (3.1)	0	0.65
ICU blood transfusion	24 (37.5)	27 (43.5)	0.49
Stroke	0	1 (1.5)	0.43

CBP, Cardiopulmonary bypass; ACC, Aortic cross-clamping; ICU, Intensive care unit

* Data are presented as mean±SD or n (%)

## Discussion

Although progressively higher-risk patients are referred for CABG, the mortality associated with isolated CABG surgery has declined substantially over the last several decades.^21^^, ^^6^^, ^^7^ Nevertheless, myocardial injury remains a significant issue in patients undergoing CABG.

L-carnitine is one of several agents having been investigated for the prevention of the clinical complications associated with ischemia-reperfusion injury during cardiac surgery.^10^ In our patients undergoing CABG surgery, treatment with L-carnitine had no effect on the levels of CK-MB or cTnT. These findings demonstrate the challenge and potential hazard of interpreting positive but non–statistically significant or post-hoc findings from relatively small trials. Although L-carnitine was not effective in reducing CK-MB and cTnT levels and thus myocardial injury in the broad population of patients undergoing CABG surgery, it may be effective in selected populations at particularly high risk of ischemia-reperfusion injury, including patients with acute MI, shock, or prolonged valve surgery.^10^ In addition, other preliminary data suggest that L-carnitine may have beneficial effects on ventricular and supraventricular arrhythmia in hemodialysis patients.^22^

Carnitine deficiency is the major cause of impaired metabolism, which mainly results in cardiomyopathy.^12^ Therefore, the administration of carnitine as a treatment for the deficiency is supported by a considerable amount of evidence.^13^^-^^15^ Our patients may have had no deficiencies, and it is possible that the L-carnitine effects on cardiac biomarkers in our study were associated with this matter. 

Any increase in CK-MB and cTnT levels after CABG surgery is suggestive of myocyte necrosis, and higher levels of these enzymes are likely to be associated with worse outcomes.^23^^, ^^24^

In this population of intermediate- to high-risk patients undergoing CABG surgery, L-carnitine did not reduce CK-MB and cTnT levels. Similar results were observed with cariporide, a specific inhibitor of the sodium-hydrogen exchanger in the large GUARDIAN trial.^25^ No reduction in death or MI was observed with cariporide compared with placebo after 36 days. However, the highest dose of cariporide was associated with a reduction in death or nonfatal MI in patients undergoing CABG surgery (relative risk, 0.75; 95% confidence interval, 0.59-0.97).^25^ So one of the reasons why L-carnitine did not have effects on enzyme levels in our study may be related to the low dosing regimen administrated.

A meta-analysis of 5 trials, including 4043 patients undergoing CABG surgery, revealed a considerable beneficial effect of purine nucleoside acadesine on the combined outcome of cardiovascular death, MI, or stroke (odds ratio, 0.73; 95% confidence interval, 0.57-0.93; p value = 0.01).^26^ A subsequent randomized clinical trial in 2698 patients failed to demonstrate a reduction in MI with acadesine; however, among patients with perioperative MI, 2-year mortality was substantially lower in those receiving acadesine.^27^ This is a promising area that deserves further study.

Our results, which were dissimilar to the findings of previous studies, may be explained by several factors and limitations. This trial enrolled intermediate- to high-risk patients undergoing isolated CABG with cardiopulmonary bypass. Some patients at the highest risk of ischemia-reperfusion injury during CABG surgery, including those with acute MI, shock, or emergency surgery, those undergoing longer operations with concomitant valve surgery, and those with more advanced renal disease, were not included in this study. The effect of L-carnitine in other populations, particularly those at higher risk of ischemia-reperfusion injury, might be different. In addition, we applied a specific dosing regimen of L-carnitine (3 g daily) based on similar studies and L-carnitine oral bioavailability. Although the absorption and plasma half-life of L-carnitine suggests that adequate levels were achieved, the intracellular kinetics of L-carnitine are not well understood and it is possible that a different dose, different dosing strategy, and duration would produce different results.^28^^-^^30^

The mechanism of myocardial injury following CABG surgery may differ from L-carnitine role as a radical scavenger and is known to be a cofactor for free fatty transportation to the mitochondria and ATP production.^10^

Although the follow-up in our study was more than 99% complete, a small number of patients (n = 24) withdrew consent or for other reasons were unable to complete follow-up.

Another reason for our results may be related to our sample size. We would strongly recommend that similar trials be repeated with larger sample sizes and different dosing regimens.

## Conclusion

Our results demonstrated that among intermediate- to high-risk patients undergoing CABG surgery, L-carnitine (3000 mg/d, given from 48 hours before surgery and continued for 2 days afterward) did not reduce CK-MB and cTnT as biomarkers of cardiac injury during CABG surgery. Myocardial injury remains a significant problem following CABG surgery. Effective therapies to reduce perioperative morbidity and mortality are needed but remain elusive.

## Notes:

***This paper should be cited as:*** Dastan F, Talasaz AH, Mojtahedzadeh M, Karimi AA, Salehiomran A, Bina P, Jalali A, Aghaie Z. Potential Effect of L-Carnitine on the Prevention of Myocardial Injury after Coronary Artery Bypass Graft Surgery. J Teh Univ Heart Ctr 2015;10(2):74-79.
